# The Duality of Caspases in Cancer, as Told through the Fly

**DOI:** 10.3390/ijms22168927

**Published:** 2021-08-19

**Authors:** Caitlin Hounsell, Yun Fan

**Affiliations:** School of Biosciences, University of Birmingham, Birmingham B15 2TT, UK; CAH618@student.bham.ac.uk

**Keywords:** caspase, cancer, tumourigenesis, *Drosophila*, apoptosis, non-apoptotic function

## Abstract

Caspases, a family of cysteine-aspartic proteases, have an established role as critical components in the activation and initiation of apoptosis. Alongside this a variety of non-apoptotic caspase functions in proliferation, differentiation, cellular plasticity and cell migration have been reported. The activity level and context are important factors in determining caspase function. As a consequence of their critical role in apoptosis and beyond, caspases are uniquely situated to have pathological roles, including in cancer. Altered caspase function is a common trait in a variety of cancers, with apoptotic evasion defined as a “hallmark of cancer”. However, the role that caspases play in cancer is much more complex, acting both to prevent and to promote tumourigenesis. This review focuses on the major findings in *Drosophila* on the dual role of caspases in tumourigenesis. This has major implications for cancer treatments, including chemotherapy and radiotherapy, with the activation of apoptosis being the end goal. However, such treatments may inadvertently have adverse effects on promoting tumour progression and acerbating the cancer. A comprehensive understanding of the dual role of caspases will aid in the development of successful cancer therapeutic approaches.

## 1. Introduction

Cancer was responsible for the death of an estimated 10 million people worldwide in 2020, making it one of the top leading causes of death in over half of countries globally [1]. Further, the cancer incidence and mortality are increasing year on year, which is, in part, a consequence of growing population ages, prevalence of risk factors and changing socio-economic conditions around the world [1]. Therefore, the impact cancer has on patients, families and communities is phenomenal and will only increase. The great need for more efficient diagnoses, better prognoses, treatment options and survival rates has fuelled cancer research for over a century.

Cancer arises in consequence of an imbalance between cell proliferation and cell death, which is frequently driven by innate biological pathways malfunctioning. This allows healthy cells to transform into malignant tumour cells and take up residency within the body. With the advancement of technologies and model systems there has been a rapid increase in knowledge and understanding of all aspects of tumourigenesis. Each stage of tumourigenesis relies on fundamental biological mechanisms and pathways from the initiation, transformation, invasion, extravasation, to the metastasis of tumour cells. Hanahan and Weinberg highlighted “The Hallmarks of Cancer”, referring to the key biological processes that must occur for a cell to become tumourigenic, including apoptotic evasion, sustained proliferative signalling, transformation, induction of angiogenesis, evasion of growth suppressors, activation of invasion and metastasis, abnormal metabolic pathways, and evasion of the immune system [2,3]. Dysregulation of numerous protein families has been linked to these “hallmarks”, including the dysregulation of cysteine-aspartic proteases, also known as caspases [4].

Apoptosis, the main form of programmed cell death (PCD), is triggered by activation of the caspase family [5,6,7]. Tumour cells frequently evade apoptosis to promote cell growth and to prevent the removal of genomically unstable tumour cells. It is therefore unsurprising that many cancers present with altered apoptotic responses. In addition to their apoptotic role, caspases have been linked to various other aspects of tumourigenesis, including (1) increased proliferation; (2) metastasis; (3) promotion of inflammation; and (4) the avoidance of immune destruction. Caspase function, outside apoptosis, is a newly developing body of research which has great implications for how we perceive the origins of caspases and the role they hold within organisms. This review focuses on the exciting recent research conducted in *Drosophila*, a model organism with advantages of genetic manipulation, and discusses the dual nature of caspase function in tumourigenesis, highlighting both the anti-tumourigenic and pro-tumourigenic roles they play.

## 2. Caspases as Drivers of Apoptosis and Beyond

Caspases are a family of highly specialized cysteine proteases which specifically cleave their substrates after an aspartate residue [8,9]. There are two classes of caspases, inflammation caspases and apoptotic caspases. Apoptotic caspases can be further broken into two groups, initiator (or apical) caspases and executioner (or effector) caspases, each with different structures and functions. Initiator caspases have a long N-terminal domain containing a death-effector domain (DEC) or caspase-recruitment domain (CARD) motif, followed by one large (p20) and one small (p10) subunit. In contrast, executioner caspases have a short or even no N-terminal prodomain in addition to the p20 and p10 subunits. Caspases were first identified in the nematode *C. elegans* in the 1980s when it was uncovered that mutations in the *ced-3* and *ced-4* genes prevented the majority of developmental cell death [10,11]. It was later shown that Ced-3 is a cysteine protease responsible for the cell death execution and Ced-4 is an adaptor protein required for the activation of Ced-3 [12,13,14]. Due to the fundamental role caspases play in the induction of apoptosis, it is unsurprising that they are well conserved across the metazoan including in *C. elegans*, *Drosophila*, and mammals [5,11,12]. There are at least fourteen caspases in humans and seven in *Drosophila*, three initiator caspases (Dronc, Dredd, Dream/Strica) and four executioner caspases (DrICE, Dcp-1, Decay, Damm) (Table 1).

**Figure 1:**
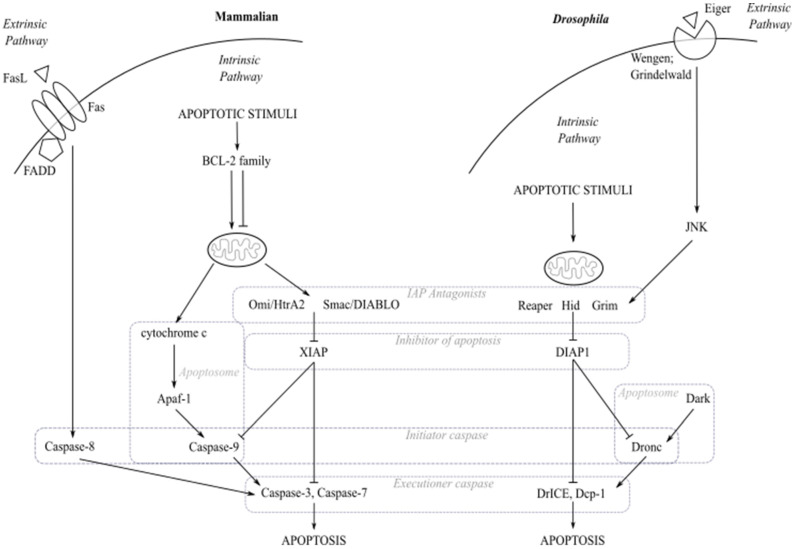
The core apoptosis pathway in mammals and *Drosophila*. During activation of the intrinsic apoptosis pathway in mammalian cells, apoptotic stimuli cause expression of pro-apoptotic BCL-2 family members resulting in the perturbation of the mitochondrial outer membrane and release of small molecules including cytochrome c, Omi/HtrA2 and Smac/DIABLO. Cytochrome c binds to Apaf-1 forming the apoptosome which recruits the pro-caspase-9 leading to its activation. Meanwhile, Omi/HtrA2 and Smac/DIABLO act as IAP antagonists to free caspases from the inhibition of apoptotic proteins such as XIAP. Active caspase-9 cleaves and activates caspase-3 and -7, allowing execution of apoptosis. The mammalian extrinsic apoptosis pathway is activated upon ligand binding to death receptor, e.g., Fas. Adaptor protein FADD recruitment to Fas forms the death-inducing signalling complex (DISC) which binds and activates caspase-8. Active caspase-8 cleaves and activates caspase-3 and caspase-7, allowing execution of apoptosis. In the intrinsic apoptosis pathway in *Drosophila*, apoptotic stimuli activate expression of pro-apoptotic proteins such as Reaper, Hid and Grim which need to localise to the mitochondria to become fully activated. These pro-apoptotic proteins release caspases from DIAP1 held inhibition. The initiator caspase Dronc then associates with the scaffold protein Dark to form an apoptosome-like complex. Active Dronc cleaves executioner caspases, DrICE and Dcp-1, which bring about apoptosis. The *Drosophila* extrinsic apoptosis pathway is activated by binding of Eiger to the death receptors Wengen or Grindelwald, resulting in activation of the JNK signalling pathway. Active JNK signalling induces expression of pro-apoptotic proteins, Hid and Reaper. The evolutionarily conserved core components in the apoptosis pathway are highlighted with the dashed line boxes.

In the absence of apoptotic stimuli, caspases exist as catalytically inactive pro-caspases (zymogens) within the cell. The core components including caspases in the apoptosis pathway are evolutionarily conserved (Table 1). Activation of the intrinsic apoptosis pathway, in response to DNA damage, intracellular stress (e.g., oxidative stress), or the extrinsic apoptosis pathway by death receptor ligand binding (e.g., Tumour Necrosis Factor (TNF)) causes pro-caspase activation via scaffold protein association (e.g., the mammalian Ced-4 ortholog, Apaf-1) or cleavage by upstream proteases [34,35] (Figure 1). In the intrinsic apoptosis pathway, upon apoptotic stimuli the expression of pro-apoptotic genes occurs, including the pro-apoptotic members of the BCL-2 protein family in mammals and the pro-apoptotic genes, such as *head involution defective* (*hid*)*, reaper* (*rpr*) and *grim* in *Drosophila*. BCL-2 family members cause perturbation of the mitochondrial outer membrane and the release of cytochrome *c* and other small pro-apoptotic proteins, such as Smac/DIABLO and Omi/HtrA2, into the cytoplasm. These pro-apoptotic proteins bind to inhibitor of apoptosis proteins (IAPs), including mammalian X-linked IAP (XIAP) and *Drosophila* DIAP1, and act as IAP antagonists which allows the release of pro-caspases from IAP inhibition [36]. Cytochrome *c* associates with Apaf-1 and binds to pro-caspase-9 through its CARD-binding motifs, forming a multi-subunit apoptosome complex [37,38,39]. Similarly, the *Drosophila* caspase-2/-9 ortholog Dronc, released from DIAP1-mediated inhibition, associates with Dark, the *Drosophila* Apaf-1 ortholog, through N-terminal CARD motif binding forming an apoptosome-like structure [37]. Apoptosome formation facilitates initiator caspase (e.g., caspase-9 and Dronc) autocatalysis and activation. The executioner caspases (e.g., mammalian caspase-3 and caspase-7, *Drosophila* DrICE and Dcp-1) are cleaved by the active initiator caspases [17]. Conversely to the intrinsic apoptosis pathway, the extrinsic apoptosis pathway, which exists in both mammals and *Drosophila*, initiates outside the cell [40]. In mammals, extracellular ligands, e.g., TNF or FasL, bind to death receptors, e.g., TNFR or Fas, on the cell surface causing receptor clustering and recruitment of adaptor proteins to the receptor [41,42]. Adaptor proteins, for example Fas-associated death domain-containing protein (FADD), bind and activate caspase-8 via the formation of the death-inducing signalling complex (DISC). The executioner caspases, caspase-3 and caspase-7, are cleaved and activated by active caspase-8. In *Drosophila*, Eiger (Egr), the sole *Drosophila* TNF ortholog, binds to its receptors Grindelwald (Grnd) or Wengen (Wgn), resulting in activation of the c-Jun N-terminal Kinase (JNK) pathway [30,31,32,33]. Active JNK induces expression of the pro-apoptotic proteins, Hid and Reaper, and subsequent caspase activation. Irrespective of activation mechanism, activated executioner caspases are responsible for the regulation of numerous biological pathways to bring about the distinct morphological changes observed during apoptosis and subsequent cell death, via the proteolytical cleavage of downstream proteins.

Intriguingly, for proteins with such a lethal nature, caspases are abundant within the cell. Therefore, their activation needs to be extremely tightly controlled to prevent rampant unwanted apoptosis with devastating consequences. Post-translation modification (PTM), proteolytic cleavage, IAP held inhibition and adaptor proteins act as defence mechanisms against this rampant caspase activation [43]. In doing so, caspase PTM creates an “all-or-nothing” activation mechanism by which apoptosis can only occur if an activation threshold is reached [44]. Given the fundamental role caspases play in maintaining tissue homeostasis and ensuring organism integrity through apoptosis, via the removal of damaged or dangerous cells, it is unsurprising that they are well conserved across the metazoan.

The evolutionary conserved roles of caspases have established the traditional view that caspases act predominantly as executors of cell death. A conserved role for caspases in inflammation is also well-established. Inflammatory caspases (e.g., caspase-1, caspase-4, caspase-11) play key roles in the innate immune response by inducing pyroptosis, to prevent pathogen replication, or by binding directly to microbial components in the cases of caspase-4, -5 and -11 [45]. The first report of inflammatory caspases was mammalian interleukin-1 β-converting enzyme (ICE), now termed caspase-1, which processes precursor interleukin-1 β into mature interleukin-1 β and shares 28% sequence homology with Ced-3 [13,46,47,48]. Although there is no *Drosophila* caspase-1 ortholog, caspases do play a role in the immune response within *Drosophila*. For example, Dredd, the *Drosophila* ortholog of the initiator caspase-8, activates Relish, a NF-kB transcription factor, in the immune deficiency (Imd) pathway [49].

Recent research paints a complex picture of caspase activity, outside their established apoptotic and inflammatory roles, with caspase activity being involved in a wide range of other fundamental cellular processes, including development, aging, proliferation, differentiation, and neuronal plasticity [50,51,52,53,54,55,56]. The collection of non-apoptotic caspase functions have been termed caspase-dependent non-lethal cellular processes (CDPs) [44]. To date more than 50 CDPs have been found [44]. One of the common features of CDPs is the involvement of cytoskeletal remodelling and cellular reshaping, as seen in dendritic pruning of *Drosophila* sensory organs and *Drosophila* spermatid terminal differentiation [57]. The exact roles of caspases in CDPs are largely unclear, and different CDPs might utilize caspases in distinct functional manners. Caspase involvement in CDPs is frequently regulated in a spatiotemporal manner to maintain sublethal caspase activity levels, preventing activation of the full-blown apoptotic cascade [58]. This highlights the importance of caspase regulation and activation thresholds. Interestingly, recent studies have reported roles for CDPs in almost every aspect of tumourigenesis, including cell proliferation, differentiation, migration and invasion [2,3,4], raising the question of what the primary function of caspases is. With a more complex picture of caspases in mind, it is easy to understand how they play intriguing and sometimes paradoxical roles in various diseases including in cancer. Further, due to their involvement in key cellular processes, caspases are uniquely situated to play a dual role in cancer, acting both as anti-tumourigenic and pro-tumourigenic agents. In this review, we discuss the recent findings from *Drosophila* studies which highlighted various apoptotic and non-apoptotic caspase roles that have increased our understanding of the dual role caspases play in tumourigenesis.

## 3. The Anti-Tumourigenic Roles of Caspases

The most prevalent anti-tumourigenic function of caspases is their ability to induce apoptosis. This functions as an intrinsic tumour suppressor, working as a defence mechanism by activating cell suicide to prevent harm from the genomically unstable cells, which harbour tumourigenic potential. It is unsurprising that circumventing this effective tumour suppressive mechanism is a defining trait of cancer [2,3]. Considering that every stage of tumourigenesis has the potential to activate the apoptosis pathway, the necessity for apoptotic evasion is obvious. Concomitantly, mutations resulting in the impairment of the apoptotic machinery are widely found in numerous forms of cancer, including follicular lymphoma, diffuse large B-cell lymphoma (DLBCL), acute myeloid leukaemia (AML), colorectal carcinomas and head and neck squamous cell carcinomas (HNSCC) [54,59,60,61,62]. In addition to the suppression of the apoptotic machinery, allowing tumour development, apoptosis can be blocked to facilitate specific oncogenic stages. For instance, during the epithelial-mesenchymal transition (EMT) cancer cells suppress anoikis, a specialized form of apoptosis which occurs during cell shedding when cells lose extracellular matrix contact, to promote metastasis [63]. In addition to these well-established anti-tumourigenic caspase functions, new studies using *Drosophila* model systems highlight a wider potential for caspases in the prevention of tumour formation, acting not only as regulators of cell death but also as regulators of cell fate [56,64].

### 3.1. Caspase-Regulated Necrosis Inhibits Tumourigenesis

Necrosis, a form of premature cell death, is characterised by cell swelling and rapid permeabilization and rupture of the cell membrane [65]. This leads to spillage of the cellular contents, including damage-associated molecular patterns (DAMPs) which can trigger an inflammatory response [66]. Usually, necrosis is initiated by extracellular factors, including cytokines, physical temperature, cell trauma, or pathogens. Recent studies have revealed that some types of necrosis are regulated therefore can be potentially managed therapeutically. One of the best studied regulated necrosis is necroptosis, which is induced by the receptor binding of TNF in mammals via a molecular mechanism distinct from apoptosis [67,68]. Active caspase-8 cleaves mammalian serine/threonine-protein kinases (RIPKs), preventing the formation of the necrosome, a complex of RIPK1, RIPK3, and MLKL, a Mixed Lineage Kinase Domain Like Pseudokinase, leading to membrane rupture once activated [69,70]. When caspase-8 is deficient or inhibited, necrosome formation occurs, resulting in the morphological changes observed during necrosis and ultimately cell death. Intriguingly, TNF can induce apoptosis or necroptosis in a context-dependent fashion, hinting at a complex interplay between these two distinct forms of programmed cell death. Open questions remain as to the factors that influence the decision between different cell death pathways and how this occurs in an intact organism in vivo.

A recent study in *Drosophila* has shed light on the roles of caspases in connecting apoptosis and necrosis [64]. In the *Drosophila* eye, expression of Egr, the *Drosophila* TNF, induces Hid-dependent apoptosis via activation of the c-Jun N-terminal Kinase (JNK) stress-response pathway. However, in the absence of the executioner caspases DrICE and Dcp-1 or expression of P35, an inhibitor of DrICE and Dcp-1, non-apoptotic cell death is induced in a Dronc-dependent manner. This cell death has been characterised to be necrosis. Additionally, it was found that loss-of-function of Basket (Bsk), the *Drosophila* JNK ortholog, or Tak1, an upstream kinase of JNK, was sufficient to block Egr-induced necrosis. For this Dronc-dependent necrosis to occur, both executioner caspase inhibition and JNK signal activation must occur. Therefore, Egr and JNK signalling have both pro-apoptotic and pro-necrotic functions and caspases play critical roles in governing the decision between these two pathways.

**Figure 2:**
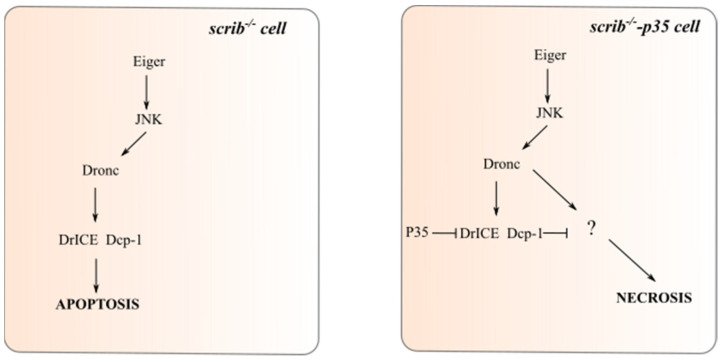
Necrosis functions as an apoptosis fail-safe mechanism in preventing tumour development. *Scrib* mutant (*scrib^-/-^*) cells are removed from the epithelial tissue via Egr/JNK-induced apoptosis to prevent tumourigenesis. When apoptosis is defective or blocked, e.g., via expression of the executioner caspase inhibitor P35, *scrib^-/-^* cells instead undergo Dronc-mediated necrosis. How Dronc activates necrosis is not yet known, indicated by the question mark.

*Drosophila* has been widely used to model and study cancer, which has been reviewed elsewhere [71,72,73,74]. Intriguingly, the Egr/JNK-mediated necrosis pathway has tumour suppressive functions. *Scribble* mutant (*scrib^-/-^*) epithelial cells lose the apical-basal cell polarity and are commonly used as an oncogenic model in *Drosophila* studies [75,76,77]. Under normal conditions, *scrib^-/-^* cells in a wild-type background are removed via Egr/JNK-mediated apoptosis to prevent rampant cell proliferation and tissue overgrowth. In *scrib^-/-^* cells in which apoptosis is blocked, e.g., by expression of P35, overgrowth is not seen due to compensatory activation of necrosis which functions as an apoptosis fail-safe mechanism (Figure 2). The cooperation observed between apoptosis and necrosis in this oncogenic model highlights a key two-layered defence mechanism to prevent tumourigenesis [64]. Notably, key components downstream of Dronc and JNK activity are still unknown. What effects caspases (e.g., Dronc, DrICE, Dcp-1) have on the decision factors that influence the apoptosis versus necrosis switch remain elusive. The idea that caspases can activate cell death, via necrosis, within tumour cells that have evaded apoptosis is intriguing and requires further investigation.

Interestingly, although Egr/JNK-mediated necrosis requires catalytically active Dronc, non-catalytic activities of Dronc have also been linked to necrosis [64,78]. For example, Dronc is required for p53-dependent necrosis, independent of its catalytic activity, during *Drosophila* spermatogenesis [78]. In this context necrotic inhibition leads to tissue hyperplasia. Notably, tumour necrosis is a common feature of many solid tumours. It is associated with hypoxia, inflammatory responses and angiogenesis and carries a poor patient prognosis [79]. Greater understanding and exploration of the mechanisms and consequences of necrosis in cancer will help provide answers and aid in the development of new therapeutic interventions.

### 3.2. Caspase-Dependent Elimination of Pre-Malignant Cells by Cell Competition

**Figure 3:**
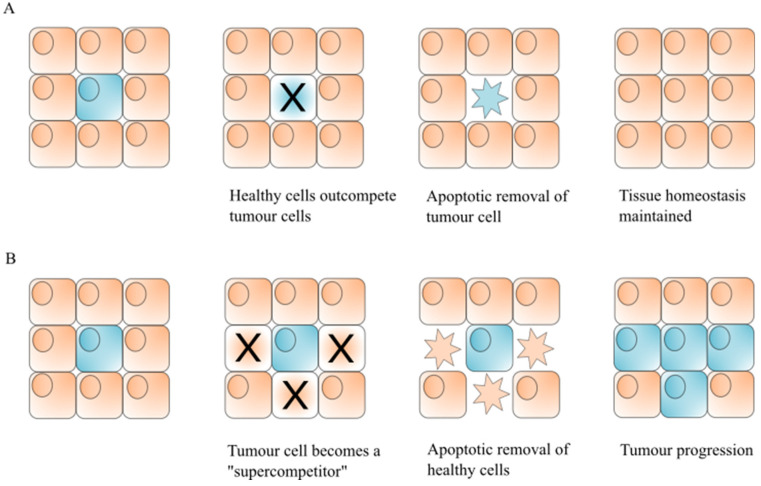
Tumour suppressive and promoting roles of cell competition. (**A**) Healthy cells (orange) have a greater cell fitness than the pre-malignant tumour cell (blue) and induce competitive cell death, removing the tumour cells via apoptosis. This acts as a tumour suppressive mechanism, preventing expansion of tumour cells and maintaining tissue homeostasis. (**B**) Tumour cells gain “supercompetitor” status through mutations, enabling the tumour cell (blue) to outcompete the healthy cells (orange). The healthy cells are removed by apoptosis allowing expansion of the tumour cell population and tumour progression.

Cell competition is used by organisms as a surveillance method to maintain tissue homeostasis and ensure organism integrity by monitoring cell fitness [80,81,82,83]. During this process, suboptimal but still viable cells (termed “losers”) are outcompeted by their “winner” neighbouring cells and subsequently eliminated by caspase-dependent apoptosis. The cell “loser” status is determined by differences from surrounding cells. For example, “loser” status can be determined by differential cell proteotoxic stress, therefore it is proposed that cells can use proteostasis as a protection mechanism against competitive cell death [84,85]. Genetic studies in *Drosophila* pioneered the field of cell competition and have uncovered both tumour suppressive and promoting roles of cell competition (Figure 3).

Research into cell competition has highlighted various mechanisms leading to differential cell fitness, dependent on the genetic backgrounds of the mixed cell populations [86,87]. Cell competition was initially observed when epithelial cells heterozygous for the *Drosophila* ribosomal protein (Rp) genes, *Rp^+/-^* or *Minute* (*M^+/-^*), were mixed with wild-type cells (*Rp^+/+^*) [88]. Although *Rp^+/-^* animals are viable, they develop more slowly compared to wild-type animals. The suboptimal *Rp^+/-^* cells are removed from mosaic epithelial tissue at interfaces with surrounding wild-type cells. Therefore, cell competition requires a heterogeneous background as differences between cells are crucial for distinction between “winners” and “losers”. Elimination of *Rp^+/-^* cells within a wild-type background occurs by JNK-dependent apoptosis [89]. In such a situation, cell competition is a mechanism of cell surveillance, to ensure tissue integrity. A recent study in *Drosophila* further showed that aneuploidy cells with chromosomal anomalies are frequently outcompeted during development because they have an altered number of ribosome genes [90]. Therefore, ribosome protein levels can act as readouts of aneuploidy cells leading to their removal by cell competition to prevent potentially disastrous consequences such as cancer.

In addition to its function in preserving tissue integrity and health, cell competition acts directly as a tumour suppressive mechanism to remove pre-malignant cells. This tumour suppressive role was initially observed when *scrib^-/-^* mutant cells were found to be outcompeted by their surrounding wild-type cells (Figure 2) [81,91]. *scrib^-/-^* cells have oncogenic potential as hyperplasia is seen in *Drosophila* epithelial tissues which contain only *scrib^-/-^* cells. However, in mosaic tissues containing *scrib^-/-^* clones, the oncogenic *scrib^-/-^* cells are kept in check by their wild-type neighbours, through cell competition [81,91]. Similarly, cell competition removes cells with other aberrant tumour suppressor genes in *Drosophila*, including other components regulating cell polarity, e.g., lethal giant larvae (Lgl) and discs large (Dlg), components of the endosomal sorting complex ESCRT-II subunit, e.g., Vps25, and key regulators of endocytosis, e.g., Rab5 [87,92,93].

Another well-studied mechanism leading to cell competition depends on the protein level of Myc, a family of transcription factors responsible for growth regulation, ribosomal biogenesis, cell proliferation and organism size, which are prominent oncogenes [94]. The role of Myc in cell competition was first reported in *Drosophila* [95]. Expression of dMyc, the *Drosophila* ortholog of mammalian c-Myc, in clones causes the competitive elimination of surrounding wild-type cells by apoptosis. Interestingly, JNK signalling only plays a minor role in this process. Therefore, upregulation of Myc generates “supercompetitor” cells which outcompete their neighbours [96]. This can be pro-tumourigenic, causing clonal expansion of oncogenic cells, while ensuring the elimination of the healthy surrounding tissue.

Caspase-dependent apoptosis mediates cell competition. In *Drosophila*, expression of P35 or DIAP1, inhibitors of caspases, or the removal of pro-apoptotic genes, *hid*, *grim* and *reaper*, protects the *Rp^+/-^* cells from competitive cell elimination. However, unlike the uncompetitive apoptosis, elimination of *Rp^+/-^* cells is not blocked by loss of the major initiator caspase Dronc. Instead, such competitive cell death can be activated redundantly by several initiator caspases including Dronc and Dream/Strica, as only co-expression of *dronc* and *dream/strica* RNAi constructs is sufficient to prevent competitive cell death [89].

Notably, recent studies have revealed that the tumour suppressive role of cell competition extends across vertebrates and bares likeness to the epithelial defence against cancer (EDAC) witnessed at the initial stages of tumourigenesis in mammalian cells [97]. Additionally, cell competition acts as a tumour suppressor in the thymus to protect the T-cell progenitor population and prevent T-cell acute lymphoblastic leukemia (T-ALL) [98]. Here, cell competition facilitates the replacement of thymus resident progenitors by the younger bone-marrow derived progenitors. The exact roles of various initiator and effector caspases remain to be seen in various cell competition processes in mammals. Nevertheless, cell competition highlights another example of an inherently tumour suppressive mechanism that can be exploited by tumours with caspases lying as regulators of the final stages of this process.

### 3.3. Caspases Regulate Stem Cells Preventing Tumourigenesis

Stem cells have self-renewal capabilities and can acquire multiple cell fates. There are multiple forms of stem cells, namely embryonic stem cells (ESCs), with unlimited differentiation potential (totipotent or pluripotent), and somatic stem cells, which have self-renewal capabilities but with restricted differentiation potential dependent on their cell derivative [55]. Interestingly, caspases exhibit non-apoptotic roles regulating multiple aspects of stem cell properties including proliferation, maintenance and differentiation [55,99]. The underlying mechanisms of these caspase functions await to be further explored in order to understand their physiological and pathological relevance. Recent studies using the adult *Drosophila* midgut, functionally equivalent to the mammalian small intestine, have led to some interesting insights [50,56].

The *Drosophila* midgut contains intestinal stem cells (ISCs) which are responsible for the continual renewal of the gut epithelium [100,101]. The ISC undergoes asymmetric cell division with self-renewal and producing an enteroblast (EB), an intermediate progenitor cell. The EB then differentiates into either an absorptive enterocyte (EC) or, much less frequently, a hormone-producing enteroendocrine cells (EE) [102]. A recently developed genetic reporter of the initiator caspase Dronc revealed that it activates in both ISC and EB cells in the midgut [103]. Subsequent studies further showed that conditional knockout of Dronc in both ISCs and EBs or RNAi knockdown of Dronc specifically in EBs causes increased cell proliferation leading to hyperplasia as well as promotes differentiation of EBs into ECs and EEs [50,56]. Moreover, the catalytic function of Dronc, but not the apoptotic cascade, is required in these processes. Therefore, the non-apoptotic Dronc activity is required to prevent aberrant proliferation and differentiation of intestinal progenitor cells including both ISCs and EBs. Notably, deficiency of the mammalian ortholog of Dronc, caspase-9, in intestinal precursor cells results in increased proliferation and abnormal differentiation [104]. Thus, caspases can regulate cell fate and act as a line of defence from aberrant proliferation and poor differentiation by keeping cells under check. Similarly, caspases can act anti-tumourigenically by causing cells to differentiate, thus removing their proliferative potential, and preventing rapid tumour growth [104]. Further studies using the *Drosophila* midgut model system may help identify key molecules and signalling pathways that mediate the tumour suppressive roles of caspase in intestinal progenitor cells.

### 3.4. Caspase Inhibition of Irradiation-Induced Cell Migration (ICM)

**Figure 4:**
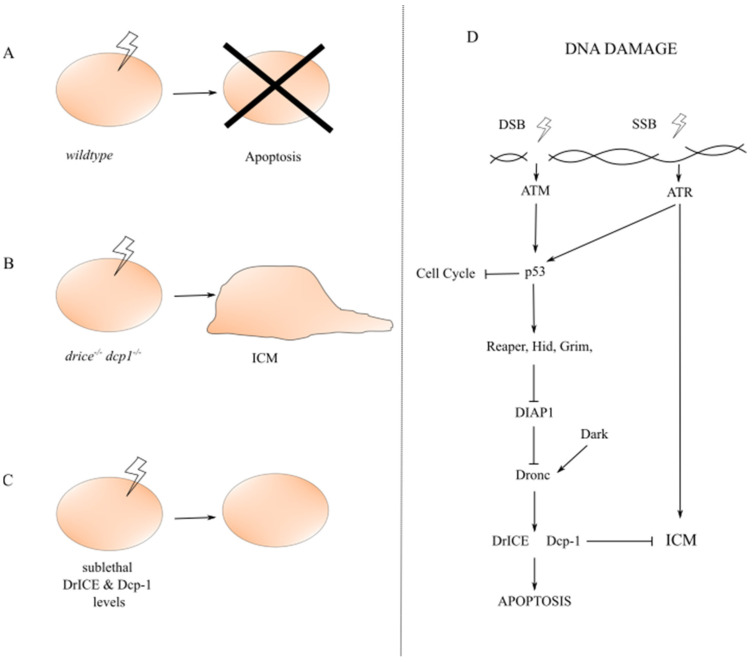
Irradiation-induced cell migration (ICM). (**A**) In wild-type cells, irradiation activates the apoptotic pathway in response to DNA damage. (**B**) In caspase-compromised, e.g., *drice^-/-^ dcp1^-/-^* cells, irradiation induces ICM. (**C**) In cells with sublethal levels of DrICE and Dcp1, the ICM is suppressed. (**D**) DNA damage, in the form of double-strand break (DSB) or single-strand break (SSB), is induced by radiation. DSBs and SSBs activate p53-dependent cell cycle arrest or, if the damage cannot be repaired, apoptosis, which is mediated by the kinase ATM or ATR. The kinase ATR, but not ATM, is required for ICM. Activation of executioner caspases, DrICE and Dcp-1, to a sublethal level inhibits ICM.

Metastatic cancer is extremely aggressive, with patients having worse prognosis and lower overall survival rates [105]. Tumour cells which gain the ability to migrate from their primary site, invade new tissues and set up residency are responsible for cancer metastasis. Recent research using *Drosophila* has identified a novel anti-tumourigenic role for low levels of caspase activation in protection against unwanted invasion and cell migration [106]. Irradiation-induced DNA damage activates the p53-dependent apoptotic pathway and mass apoptosis in developing *Drosophila* larval epithelial tissue. The executioner caspase DrICE is crucial for irradiation-induced apoptosis within this setting. Intriguingly, DrICE and Dcp-1 null mutant cells (*drice^-/-^ dcp-1^-/-^*), cells with compromised caspase function, do not undergo apoptosis post-irradiation, instead they gain migratory ability and invasive potential, in a process termed “irradiation-induced cell migration” (ICM) (Figure 4). Importantly, *drice^-/-^ dcp-1^-/-^* cells delaminate basally and migrate out of their native tissue in a self-driven manner which is indispensable of other cellular process such as phagocytosis. However, compromised caspase function is required in the invaded tissue to allow the invasion of caspase deficient cells post-irradiation, indicating a potential role of caspase-mediated cell non-autonomous signals to ensure invasive receptivity. Also, not all *drice^-/-^ dcp-1^-/-^* cells migrate therefore pro-migratory factors other than compromised caspases must be involved. Although the caspase substrates mediating ICM have not been identified yet, a main contributor to this is the remodelling of the cytoskeleton. The Rho GTPases, a family of small G-proteins, are heavily involved in actin dynamics, cytoskeletal remodelling, and cell motility. Indeed, knockdown or inactivation of the small GTPases such as Rho1, Rac1, Rac2 and Cdc42 reduces the number of observed migratory cells. Additionally, components of the extracellular matrix are important for cell movement and accumulation of matrix metalloproteinase 1 (MMP1), which breaks down the extracellular matrix, is observed in some but not all *drice^-/-^ dcp-1^-/-^* cells. This may suggest a transient requirement of MMP1 and highlight remodelling of the extracellular matrix as an important factor of ICM.

Intriguingly, the process of ICM bares many similarities to the EMT, which occurs during normal development and in the metastatic expansion of tumours [106]. Specifically, there are significant morphological similarities (e.g., basal delamination of cells), as well as behavioural and molecular features. Therefore, sublethal levels of executioner caspases, DrICE and Dcp-1, can act as protectors against unwanted cell migration and inhibit ICM. Additionally, distinct DNA damage response signalling molecules appear to mediate and distinguish the cellular responses of apoptosis versus ICM (Figure 4) [106]. For example, Ataxia Telangiectasia and Rad3-related (ATR), the single-strand DNA break (SSB)-activated kinase, is required for ICM. In contrast, Ataxia Telangiectasia Mutated (ATM), the kinase activated by the double-strand DNA break (DSB), and p53 are required for apoptosis but not ICM. These suggest that different types of DNA damage may determine the cellular migratory potential, which needs to be further investigated. Notably, spontaneous cell migration without irradiation also occurs in caspase mutant cells, hinting at the role of ICM within cancer as many tumour cells have compromised caspase function. It has been proposed that the sublethal roles of caspases, including such an anti-tumourigenic and pro-survival mechanism, are the house-keeping functions of caspases [44]. More research is required to better understand these.

## 4. The Pro-Tumourigenic Roles of Caspases

In addition to their anti-tumourigenic roles, caspases can also act to promote tumourigenesis within pre-malignant and malignant cells. Intrinsic tumour suppressor mechanisms can be exploited during tumourigenesis to facilitate oncogenic transformation. For example, the role necrosis plays in tumourigenesis is not straightforward. In addition to acting as a defence mechanism, patients with large necrotic regions within their tumours (i.e., known as necrotic cores) have a much worse outcome and lower survival rate. This occurs due to necrosis-induced acute and chronic inflammation resulting from the necrotic release of DAMPs that promote inflammation, release tumour-promoting cytokines and, ultimately, promoting tumour progression [107,108]. However, the role caspases play in pro-tumourigenic necrosis needs to be further explored.

Similarly, cell competition can also be highjacked by cancer cells for a pro-tumourigenic gain [83]. During cell competition in mosaic tissue, loser cells are initially eliminated from the borders of clonal patches leaving the central cells intact. In tumours this can result in rampant apoptosis and JNK signalling at the tumour edges causing rampant proliferative signalling and rapid tumour expansion [80,83]. Moreover, as introduced above (Figure 3), a model of tumour “supercompetitiors” has been proposed [96]. Tumour cells gain the ability to outcompete their neighbouring normal cells resulting in the rapid removal of healthy tissue. One way this occurs is by cells gaining the ability to escape proteotoxic-induced cell competition [84]. The tumour competitive growth advantage has the potential to make tumours more aggressive, with worse outcomes. A study using *Drosophila* midgut showed that cells with mutations in the *Adenomatous polyposis coli (APC)*, a tumour suppressor gene, eliminate adjacent wild-type cells via cell competition leading to hyperplasia and tumour formation [109]. This tumour growth can be prevented by caspase inhibitors (DIAP1 or P35 expression) indicating that caspase-dependent apoptosis is solely responsible for healthy cell removal.

Furthermore, a fine balance between cell proliferation, cell death and cell differentiation needs to be achieved to maintain homeostasis within the tissue. Deregulation of this balance can have pathological consequences. In addition to the inhibitory role of caspases in stem cell proliferation as described above (see Section 3.3), dysregulation of caspases can also promote deregulation of stem cell properties and lead to tumour formation and progression by forming subpopulations of proliferative cells in tissues, that cannot differentiate normally [55].

To survive, tumour cells rely on utilising endogenous proteins and signalling pathways to fuel their growth. As crucial proteases driving cell death, caspases are often disrupted or hijacked within tumour cells to facilitate their over-proliferation, migration, tissue invasion and metastasis. Recent work in *Drosophila* has revealed some insightful examples for these.

### 4.1. Apoptosis-Induced Proliferation

The most fundamental feature of cancer cells is their ability to deregulate tissue homeostatic signalling and gain sustained proliferative potential [2,3]. They can do so by either reproducing mitogenic signals themselves or by stimulating their surrounding healthy cells to produce mitogenic signals on their behalf. The non-apoptotic roles of caspases in proliferative signalling have been revealed. Current research is aiming to answer how these roles are achieved and regulated.

**Figure 5:**
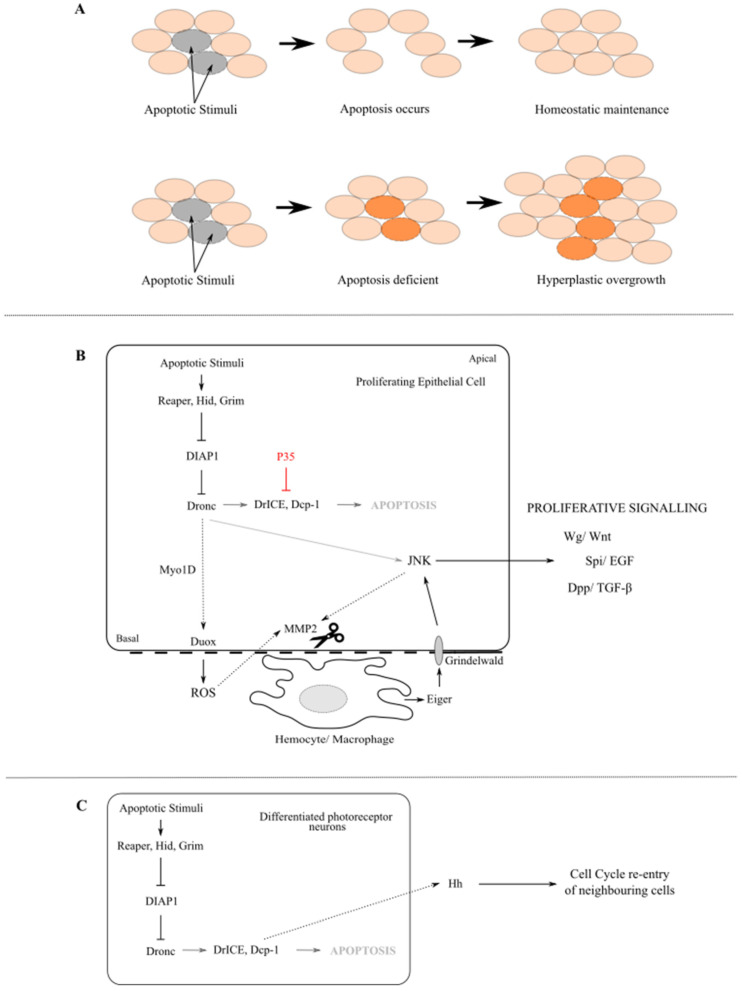
Apoptosis-induced Proliferation (AiP). (**A**) Roles of AiP in homeostatic maintenance and tumourigenesis. (**B**) Mechanism of AiP in apoptosis-deficient proliferation cells leading to tissue overgrowth. The initiator caspase Dronc coordinates apoptosis and AiP in response to apoptotic stress. P35 inhibits DrICE and Dcp-1, therefore execution of apoptosis, but not Dronc. This allows continuous AiP resulting in tissue overgrowth. Myo1D interacts with Dronc and facilitates its re-localisation to the basal plasma membrane, where Dronc regulates Duox to stimulate production of ROS. ROS and JNK activate MMP2, which disrupts the basement membrane leading to recruitment of hemocytes (macrophages). Macrophages in turn secret Eiger and activates JNK via the TNF receptor Grindelwald in epithelial cells non-cell autonomously. Activated JNK drives hyperplastic tissue overgrowth and tumourigeneisis through activation of mitogens including Wg/Wnt, Dpp/TGF-β and Spi/EGF. (**C**) Mechanism of AiP in the differentiating eye tissue. In response to apoptotic stress, the executioner caspases DrICE and Dcp-1 in dying photoreceptor neurons activate the Hh growth signals, which secrete to their neighbouring cell cycle-exited but unspecified cells and drive their cycle re-entry.

In 1970s, it was first reported that the *Drosophila* larval wing epithelial tissue can recover from X-ray radiation-induced cell death with additional cell proliferation resulting in the adult flies with normal wings [110]. It was later shown that the stress-induced apoptotic cells can stimulate their neighbouring cells to proliferate to compensate for cell loss and maintain tissue homeostasis [111,112,113]. This compensatory proliferation, later redefined as apoptosis-induced proliferation (AiP), plays a key role in tissue recovery from damage (Figure 5A). Similar phenomena have also been observed in other multi-cellular organisms including Hydra, planarians, Xenopus, newt and mouse [114,115,116,117]. Under pathological conditions when execution of apoptosis is compromised therefore the stressed cells are kept alive or “undead”, the continuously activated AiP promotes tissue overgrowth (Figure 5A). AiP is thus also a phenomenon relevant to cancer as cancer cells frequently evade apoptosis. Indeed, AiP has been shown to promote cancer development, cell invasiveness and faster tumour recurrence after radio- and chemo-therapies [118,119,120,121]. Therefore, understanding of the molecular mechanisms regulating AiP will have implications on cancer treatment.

From the initial discovery of AiP, *Drosophila* has remained the focal point for AiP research. Initial studies using *Drosophila* epithelial tissues were able to provide key insights into the AiP pathway, which requires non-apoptotic caspase activity. Two distinct mechanisms of AiP have been uncovered in epithelial tissues in *Drosophila* (Figure 5B,C). In the proliferating tissue, the initiator caspase Dronc has a non-apoptotic role to activate JNK leading to the release of mitogens including Wingless (Wg), the *Drosophila* Wnt, Decapentaplegic (Dpp), the *Drosophila* transforming growth factor-β (TGF-β), and Spitz (Spi), the *Drosophila* EGF [111,112,113,122,123]. In contrast, in the differentiating eye tissue where cells have exited the cell cycle, the executioner caspases DrICE and Dcp-1 activate the Hedgehog (Hh) signalling in the photoreceptor neurons to drive cell cycle re-entry of the neighbouring cell cycle-exited but unspecified cells [124]. These findings uncoupled the non-apoptotic roles of caspases from their apoptotic functions. However, our understanding of the mechanisms underlying these caspase functions are far from complete.

Activation of JNK signalling has been identified as a hallmark of Dronc-induced AiP in the proliferating tissue (Figure 5B). It has recently been shown that active Dronc is translocated from cytosol to the basal side of the plasma membrane, where it executes its role in AiP [125]. Although how Dronc re-localisation occurs is not yet clear, Myo1D, an unconventional myosin, is critical in this process and interacts directly with Dronc. At the plasma membrane, Dronc associates with Duox, a NADPH oxidase, and stimulates the production of reactive oxygen species (ROS), which promote AiP. Further studies revealed that ROS cause damage to the basement membrane of the epithelial tissue, via a matrix metalloproteinase MMP2, and attract hemocytes, the *Drosophila* macrophages, to the site [126,127]. The accumulated macrophages secrete the TNF ligand Egr which activates the JNK signalling pathway non-cell autonomously via its receptor Grindelwald (Grnd). Notably, ROS-mediated basement membrane disruption and macrophage recruitment are required for tissue overgrowth in various *Drosophila* neoplastic tumour models [127,128]. This suggests that non-cell autonomous activation of JNK is critical for AiP-driven tumourigenesis. Intriguingly, cell autonomous activation of JNK has also been implicated in AiP. For example, JNK is also required for the regulation of MMP2 and basement membrane damage prior to macrophage recruitment. Moreover, JNK is activated in damaged cells to trigger regeneration of epithelial tissues in response to apoptotic stress, without a clear involvement of macrophages [54,129]. Therefore, activation of JNK may occur at different stages of AiP and contribute distinctly to tumour development. Dronc, as the key caspase acting upstream of JNK, may regulate cell autonomous and non-cell autonomous activation of JNK via different mechanisms. Importantly, JNK signalling has been widely implicated in cancer development and progression [130]. However, it has been challenging to dissect its complex roles and identify therapeutic targets in cancer, partially because three JNK genes exist in mammals. Further investigation of the regulation of JNK in *Drosophila* tumour models will provide useful insights to address this.

Upstream of JNK, caspase-dependent ROS production and macrophage recruitment observed in tumourigenesis in *Drosophila* also show striking similarities to the features of the mammalian tumour microenvironment. For example, activation of tumour-associated macrophages (TAMs) driven by apoptosis has been proposed to be a key element of the “onco-regenerative niche” promoting malignant cancer [131]. Moreover, ROS are required for the recruitment of TAMs [132]. However, it remains to be seen whether functions of caspases in these cellular processes are similar to their fly counterparts.

### 4.2. Invasion and Metastasis

Metastasis poses the major challenge to the overall survival rate of cancer patients, with most cancer-related deaths occurring as direct consequence of secondary sites metastases [105]. Metastasis occurs when tumour cells disseminate from the primary tumour site and colonize a secondary site elsewhere in the body [133,134]. This process is coordinated through a complex series of biological processes, referred to as invasion-metastasis cascade. Further, the activation of invasion and metastasis is one of the ‘Hallmarks of Cancer’ [2,3]. During successful metastasis, tumour cells lose cell-cell adhesion and gain increased invasive and migratory potential during the EMT, allowing the tumour cells to invade surrounding tissue [135]. The tumour cells enter circulation either in the bloodstream or lymphatic system. Only a fraction of circulating tumour cells forms successful metastases after they successfully invade the endothelium, travel to a distinct site and undergo mesenchymal-epithelial transition (MET) [133,134,136]. Governing metastasis is a multitude of complex biological processes, a better understanding of why, how and when these aggressive metastases occur would have phenomenal patient benefits.

High levels of effector caspase activity in cancer cell lines, patient tissue and clinical data are correlated with increased invasiveness and aggression [120]. Using a *Drosophila* invasion model system, sublethal caspase activity was found to cause cell invasion [137]. A low level of caspase activity, due to expression of the inhibitor P35 or RNAi knockdown of DrICE, causes cell detachment from the basal membrane and local migration in the epithelial tissue. The migration occurs via activation of JNK signalling and expression of MMP1, which degrades the extracellular membrane [137]. The effector caspase, DrICE, is required to promote invasion. A complete loss-of-DrICE suppresses the cell invasion. Further, the DrICE-dependent invasion is mediated by the upstream initiator caspase Dronc and is apoptosis-independent because inducing cell death alone, e.g., without p35 expression, was not sufficient to cause cell migration. This suggests that, in the absence of apoptosis, tumour cells may be more likely to migrate. Therefore, apoptotic evasion, as one of the defining traits of cancers, might be intrinsically linked to cancer metastasis. Similarly, suppression of *vestigial* (*vg*), a gene required for cell proliferation in the *Drosophila* wing epithelia, induces cell migration dependent on the moderate levels of caspase activity and JNK activation [138]. Moreover, sublethal caspase activity has also been reported to trigger a developmental cell delamination process during *Drosophila* thorax fusion [139]. Therefore, caspases may possess a two-layered activity with the basal level to drive cell migration rather than apoptosis. Notably, as described above, caspases inhibit ICM in response to DNA damage (Figure 4). Hence, the roles of caspases in metastasis is complex, which likely depend on the cellular context and the nature of the stress stimuli. This has major implications for cancer therapies. Triggering cell death, through chemotherapy or radiotherapy, remains one of the standard therapeutic approaches for cancer treatment. Sublethal caspase activity or failed apoptosis can contribute to tumour recurrence and metastasis, thus unsuccessful treatments might induce the metastatic process and acerbate malignancy [137,140]. Therefore, understanding how caspase activity causes metastasis is paramount for the delivery of successful long-term treatment regimens.

## 5. Conclusions

Research in the past four decades have revealed diverse and distinct roles of caspases in normal biology. Caspase function, both apoptotically and non-apoptotically, frequently becomes deregulated during cancer formation and progression, allowing for pre-malignant and malignant cells to thrive. The emerging field of non-apoptotic caspase function has a multitude of unanswered questions, both for normal physiology and cancer. Why is caspase activity context-dependent? And what governs this? How do cells detect and distinguish the basal caspase activities versus lethal caspase levels therefore respond differently? Does the homeostatic role of caspases represent their ancient housekeeping function?

The context-dependent dual roles of caspases as both pro- and anti-tumorigenic factors, and the complex interplay between the two have serious implications for cancer therapies. A common therapeutic route is to induce apoptosis in tumour cells, e.g., via chemoradiotherapy. However, as our understanding of the dual roles that the apoptotic machinery has in tumour formation and progression increases, this common therapeutic route may need to be re-evaluated. For example, radiation can induce pro-tumourigenic stem cell properties. Recent studies in *Drosophila* have shown this is caspase-dependent and has serious implications for radiotherapy regimes [141]. Additionally, caspase inhibition can induce TNF-mediated necrosis which might pose therapeutic complications when giving anti-apoptotic agents [142].

*Drosophila* research has been at the forefront of our increased understanding of the complex role of caspases. The use of *Drosophila* model systems allows efficient in vivo genetic screens and effective drug screening [143]. This has considerable benefits for cancer research. Importantly, many of the processes first uncovered in *Drosophila*, including AiP and cell competition, are conserved and exist in mammals. Therefore, the works of *Drosophila* have direct implications for understanding cancer and in the development of novel cancer therapies.

## Figures and Tables

**Table 1:** Caspases and conserved components of the apoptosis pathway.in humans and *Drosophila*.

	Protein	Human	*Drosophila*
Apoptotic Caspases	Initiator Caspases	Caspase-2, -8, -9, -10 [15,16]	Dronc, Dredd, Dream/Strica [17]
	Executioner Caspases	Caspase-3, -6, -7 [15,16]	DrICE, Dcp-1, Decay, Damm [17]
Other Conserved Apoptosis Pathway Components	Adaptor (Scaffold) Protein in the Apoptosome	Apoptotic protease activating factor-1 (Apaf-1) [18]	*Drosophila* Apaf-1-related killer (Dark) [19]
	Inhibitor of Apoptosis Proteins (IAPs)	XIAP, Ts-IAP, cIAP1, cIAP2, ML-IAP, Neuronal Apoptosis Inhibitory Protein (NAIP), Survivin, BRUCE [20]	DIAP1, DIAP2 [21]
	IAP Antagonists	Omi/HtrA2, Smac/DIABLO, ARTS [22]	Reaper, Hid, Grim, Sickle [22]
	B-cell lymphoma 2 (BCL-2) family	25 reported members including anti-apoptotic BCL-2, BCL-xL, BCL-w, Myeloid cell leukemia 1 (MCL-1) and pro-apoptotic BCL-2 antagonist killer 1 (BAK), BCL-2-associated x protein (BAX), BCL-2-related ovarian killer (BOK) [23,24]	Buffy, Debcl [25,26,27,28]
	Death Receptors	TNF receptor superfamily with 29 reported members including TNFR1, TNFR2, Fas, TRAIL-R1 and TRAIL-R2 [29]	Grindelwald [30], Wengen [31]
	Death Receptor Ligands	TNF ligand superfamily with 19 reported members including TNFα, TNFβ, FasL and TRAIL [29]	Eiger [32,33]

## Abbreviations

AiP	Apoptosis-induced proliferation
Apaf-1	Apoptotic protease activating factor-1
ATM	Ataxia Telangiectasia Mutated
ATR	Ataxia Telangiectasia and Rad3-related
BCL-2	B-cell lymphoma 2
CDP	Caspase-dependent non-lethal cellular processe
DAMPs	Damage-associated molecular patterns
Dark	*Drosophila* Apaf-1-related killer
Dcp-1	*Drosophila* caspase-1
DIABLO	Direct Inhibitor of Apoptosis-Binding protein with LOw pI
DIAP1	*Drosophila* Inhibitor of Apoptosis Protein 1
Dpp	Decapentaplegic
DrICE	Death related ICE-like caspase
Dronc	*Drosophila* Nedd2-like caspase
DSB	Double-strand DNA break
Duox	Dual oxidase
EGF	Epidermal growth factor
Egr	Eiger
EMT	Epithelial-mesenchymal transition
FADD	Fas-associated death domain-containing protein
Grnd	Grindelwald
Hh	Hedgehog
Hid	Head involution defective
HtrA2	High temperature requirement factor A2
IAP	Inhibitor of Apoptosis Protein
ICE	Interleukin-1β converting enzyme
ICM	Irradiation-induced cell migration
JNK	c-Jun N-terminal Kinase
MET	Mesenchymal-epithelial transition
MMP	Matrix metalloproteinase
Myo1D	Myosin 1D
PCD	Programmed cell death
ROS	Reactive oxygen species
Rpr	Reaper
Smac	Second mitochondria-derived activator of caspase
Spi	Spitz
SSB	Single-strand DNA break
TAM	Tumour-associated macrophage
TGF-β	Transforming growth factor-β
TNF	Tumour Necrosis Factor
TNFR	TNF receptor
TRAIL	Tumor necrosis factor-related apoptosis-inducing ligand
TRAIL-R	TRAIL receptor
Wg	Wingless
Wgn	Wengen
Wnt	Wingless-related integration site
